# From North to South: transmission dynamics of H1N1pdm09 swine influenza A viruses in Italy

**DOI:** 10.1099/jgv.0.002174

**Published:** 2025-11-13

**Authors:** Marta Giovanetti, Eleonora Cella, Laura Soliani, Alice Prosperi, Ada Mescoli, Ambra Nucci, Carla della Ventura, Dennis Maletich Junqueira, Nídia S. Trovão, Francesco Branda, Maya Carrera, Davide Lelli, Carlo Rosignoli, Silvia Faccini, Laura Fiorentini, Flavia Guarneri, Gianguglielmo Zehender, Massimo Ciccozzi, Chiara Chiapponi, Ana Moreno

**Affiliations:** 1 Department of Sciences and Technologies for Sustainable Development and One Health, Università Campus Bio-Medico di Roma, Rome, Italy; 2 Instituto René Rachou, Fundação Oswaldo Cruz, Belo Horizonte, Minas Gerais, Brazil; 3 Climate amplified diseases and epidemics (CLIMADE) Americas, Rio de Janeiro, Brazil; 4 Burnett School of Biomedical Sciences, College of Medicine, University of Central Florida, Orlando, FL 32827, USA; 5 Istituto Zooprofilattico Sperimentale della Lombardia e dell'Emilia Romagna, WOAH Reference Laboratory for swine Influenza, Brescia, Italy; 6 Department of Biomedical and Clinical Sciences, University of Milan, Milan, Italy; 7 EpiSoMI - Centre for Epidemiology and Molecular Surveillance of Infections, University of Milan, Milan, Italy; 8 Programa de Pós-Graduação em Ciências Biológicas: Bioquímica Toxicológica (PPGBTox), Laboratório de Bioinformática e Evolução Viral, Universidade Federal de Santa Maria (UFSM), Santa Maria, Rio Grande do Sul 97105-900, Brazil; 9 Fogarty International Center, National Institutes of Health, Bethesda, MD 20892, USA; 10 Unit of Medical Statistics and Molecular Epidemiology, University of Campus Bio-Medico di Roma, Rome, Italy

**Keywords:** genomic epidemiology, H1N1, Italy, pandemic strains, swine

## Abstract

The influenza A H1N1pdm09 virus continues to pose a significant zoonotic threat, with implications for both animal and human health. Italy, which hosts one of the largest swine populations in Europe, is strategically positioned to monitor the evolution of influenza viruses in livestock. This study addresses the genetic diversity and transmission dynamics of H1N1pdm09 in Italian swine, using whole-genome sequencing and dynamic modelling of samples collected from farms across the country. Our findings indicate multiple independent introductions of H1N1pdm09 into Italy. While most were self-limiting, six distinct transmission clusters suggest localized and sustained spread across various regions. Although many introductions were contained, certain lineages demonstrated the ability to circulate within specific areas. Selective pressure analyses showed strong purifying selection across most viral genes in both swine and human hosts, with non-synonymous to synonymous substitution rate (dN/dS) ratios well below 1. The haemagglutinin gene exhibited a higher dN/dS ratio in swine (~0.28) than in humans (~0.22), indicating slightly relaxed selection in swine. Neuraminidase and non-structural proteins were similarly constrained in both hosts. This study underscores the importance of ongoing genomic surveillance to detect viral circulation and mitigate zoonotic risks. Italy’s contribution supports global influenza monitoring and reinforces the need for a One Health approach that integrates human, animal and environmental health. These insights are crucial for informing public health strategies and improving preparedness for future outbreaks.

## Data availability

The data supporting the findings of this study are available from the corresponding author upon reasonable request. This study was previously made available as a preprint on bioRxiv [Giovanetti, M., Cella, E., Soliani, L., Prosperi, A., Mescoli, A., Nucci, A., and Moreno, A. (2024). From North to South: transmission dynamics of H1N1pdm09 swine influenza A viruses in Italy. bioRxiv. https://doi.org/10.1101/2024.12.12.628126]. All data associated with the newly generated H1N1 swine genome sequences from Italy in this study can be found in Table S1.

## Introduction

Zoonotic diseases – those that originate in animals and are transmissible to humans – pose a persistent threat to global health, particularly when driven by rapidly evolving viruses such as influenza A. The rise and fall of human history have often been shaped by the appearance and dissemination of infectious diseases [1]. Among these, zoonotic diseases, which originate from animals and have the capacity to infect both animals and humans, play a pivotal role. These diseases underscore the profound implications of interspecies interactions, often acting as environments for viral recombination and ushering in new threats with potential epidemic or pandemic dimensions [2]. Within this landscape, the 2009 H1N1 pandemic (H1N1pdm09, clade HA-1A) influenza strains emerged as a significant challenge [2].

At the onset of the 2009 influenza pandemic, the H1N1pdm09 virus was identified as a unique reassortant strain, combining genetic elements from three swine influenza lineages. Notably, key segments were derived from a North American H3N2 swine virus present since the mid-1990s [3]. The H1 segment traced back to the classical swine H1N1 lineage circulating since the 1918 pandemic, while the N1 and MP segments were linked to the Eurasian swine lineage that emerged in the late 1970s. The initial human outbreak of the H1N1pdm09 virus is believed to have occurred in Mexico, as indicated by genetic and epidemiological evidence [3]. This virus’s ability to cross species barriers is a hallmark of its genetic composition, which includes elements from swine, avian and human influenza A viruses (IAVs) [4]. This characteristic has consistently posed epidemiological challenges. Over time, H1N1pdm09 has contributed internal gene segments through reassortment to the generation of novel IAVs, rather than evolving as a single lineage. These reassortant strains exhibit distinct genetic constellations and, in some cases, altered phenotypic characteristics [4].

Italy plays a prominent role in European swine production. According to Food and Agriculture Organization (FAO) estimates for 2023, Italy ranks among the top eight swine-producing countries in Europe [5]. The country maintains a diverse and specialized pig industry, with high-density farming operations particularly concentrated in northern regions such as Lombardy, Emilia-Romagna and Veneto. This makes Italy a critical setting for monitoring the dynamics of influenza viruses in swine populations [5].

In European countries, the endemic H1 swine strains circulating since the 1980s, H1avN1 (HA-1C) and H1huN2 (HA-1B), have gradually been joined by new genotypes resulting from various reassortment events, such as novel H1pdm09N2 (HA-1A) and H1avN2 (HA-1C). This has led to increased genetic and antigenic diversity within these H1 lineages [6,7], contributing to the overall genetic diversity of swine influenza viruses circulating across European pig herds [5]. In Italy, known for its robust pig-farming sector, a significant surge in the genetic diversity of swine influenza viruses has been observed over the last 10 years [8]. This period has seen the emergence of different H1N1 and H1N2 subtypes characterized by diverse gene combinations. Since 2009, Italy has consistently reported the H1N1pdm09 IAV subtype, which accounts for ~10% of the H1N1 isolates identified [9]. However, the lack of historical data on the circulation of IAV in Italian swine populations has made it challenging to trace the origins and transmission of the H1N1pdm09 strain within the country.

Given the importance of pig production in Italy, characterized by a diversified pig population and an extensive herd network, active genomic surveillance is essential. This surveillance is crucial to monitor the evolution of these viruses, particularly regarding reassortment phenomena involving human and avian influenza viruses. Such monitoring will allow for better investigation and understanding of the dynamics of swine influenza and provide crucial insights for global pandemic preparedness and response strategies.

In this study, we employed whole-genome sequencing and phylogenetic inference methods to address the existing knowledge gap regarding H1N1pdm09 swine strains circulating in Italy. Our analysis focused on influenza A viruses in swine (IAV-S) strains characterized by a full genome of H1N1pdm09 origin, revealing multiple introductions of the virus, with many resulting in self-limited infections in swine and limited onward transmission. However, we identified a few sustained transmission clusters (*n*=6) during this period. Through a comprehensive examination of these strains, including selective pressure analysis (dN/dS), we gained valuable insights into their evolutionary pathways and transmission dynamics within the country. These findings highlight the importance of continuous genomic monitoring as an essential tool for proactive health strategies and emphasize Italy’s significant role in advancing the study of influenza viruses from a One Health perspective.

## Methods

### Ethical approval

Ethical approval was not required for this study, as it involved the analysis of residual samples obtained from IAV-positive specimens (lungs, nasal swabs or oral fluids). These samples were submitted to the Istituto Zooprofilattico Sperimentale della Lombardia e dell’Emilia Romagna for diagnostic confirmation of pigs with respiratory syndrome between 2009 and 2024. The positive samples were characterized by the World Organisation for Animal Health (formerly OIE, *Office International des Epizooties*) (WOAH) Reference Laboratory for swine influenza under informed consent, which permits their use for research purposes to accelerate knowledge building and support surveillance and outbreak response efforts.

#### Collection and sequencing of H1N1pdm09 IAV in Italian pigs

Swine influenza sequences were obtained from IAV-positive samples (lungs, nasal swabs or oral fluids) submitted to the Istituto Zooprofilattico Sperimentale della Lombardia e dell’Emilia Romagna for diagnostic confirmation of pigs with respiratory syndrome from 2009 to 2024. The samples had previously been tested by real-time PCR (RT-PCR) for the M-gene and subtyped by multiplex RT-PCR [10]. Virus isolation was performed using cell cultures [10]. Whole-genome sequences were obtained from virus isolates or, if negative, from diagnostic samples with Cycle threshold (Cts) lower than 32, as described previously by Lycett *et al*. [11], using the SuperScript^®^ III One-Step RT-PCR System with Platinum^®^ Taq High Fidelity (Thermo Fisher Scientific). RT-PCR products were purified using NucleoSpin^®^ Gel and PCR Clean-up (Macherey-Nagel, Carlo Erba, Italy). Sequencing libraries were made with a Nextera XT DNA Library Prep Kit (Illumina Inc., San Diego, CA, USA) according to the manufacturer’s instructions and sequenced on a MiSeq Instrument (Illumina) using a MiSeq Reagent Nano Kit v2 in a 150-cycle paired-end run. Data were *de novo* assembled using the CLC Genomics Workbench v.11 (Qiagen, Milan, Italy) [12,13]. Genotype nomenclature was used as previously described [10]. A total of 45 genomes of the H1N1pdm09 lineage were obtained. All samples came from large commercial facilities in the Italian macro-regions: Northeast (*n*=15), Northwest (*n*=21), Central (*n*=1), South (*n*=5) and Insular (*n*=3). Complete details of all genome sequences obtained in this study are available in Table S1.

#### Phylogenetic and phylodynamic analysis of swine H1N1 in Italy and spatial analysis

In addition to the 45 whole-genome sequences generated in this study, background sequences were downloaded from the Global Initiative on Sharing All Influenza Data’s (GISAID, https://gisaid.org/publish/) EpiFlu database. All available H1N1pdm09 sequences were downloaded by gene segments (accessed on 7 June 2024). Sequence alignments were constructed for each of the six internal gene segments [polymerase basic 2 (PB2), polymerase basic 1 (PB1), polymerase acidic (PA), nucleoprotein (NP), Matrix Protein (MP) and non-structural (NS)] and the two surface antigen segments [haemagglutinin (HA) and neuraminidase (NA)] using Multiple Alignment using Fast Fourier Transform (MAFFT) [14]. Alignments were edited with AliView to remove biological artefacts [15]. IQ-TREE2 [16] was used for maximum-likelihood (ML) phylogenetic analysis of each of the eight alignments, employing the general time-reversible model of nucleotide substitution and a proportion of invariable sites (+I) as selected by the ModelFinder option. The final datasets for each segment, after removing outliers and low-quality sequences, included the following numbers of sequences: HA (*n*=510, including 45 Italian sequences), NA (*n*=522, including 45 Italian sequences), PB1 (*n*=428, including 45 Italian sequences), PB2 (*n*=459, including 45 Italian sequences), PA (*n*=481, including 45 Italian sequences), NP (*n*=464, including 45 Italian sequences), MP (*n*=554, including 45 Italian sequences) and NS (*n*=471, including 45 Italian sequences). We performed a joint estimation of phylogenetic relationships and dispersal history for each dataset separately using a time-scaled Bayesian approach with Markov Chain Monte Carlo (MCMC) method implemented in BEAST v1.10.4 [17]. A relaxed uncorrelated lognormal (UCLN) molecular clock was used, with a Skygrid population size [18] and a general-time reversible (GTR) model of nucleotide substitution with gamma-distributed rate variation among sites. The phylogeographic analysis considered the host as locations (swine and human). The location state was specified for each viral sequence, allowing the expected number of location-state transitions in the ancestral history conditional on the data observed at the tree tips to be estimated using ‘Markov jump’ counts [19], which provided a quantitative measure of asymmetry in gene flow between locations. For computational efficiency, the phylodynamic analysis was run using an empirical distribution of 1,000 trees [19], allowing the MCMC chain to run for 100 million iterations (twice, in two separate runs), sampling every 10,000. A Bayesian stochastic search variable selection (BSSVS) was employed to improve statistical efficiency for all datasets. All parameters reached convergence, as assessed visually using Tracer v.1.7.1, with all parameters achieving effective sample sizes greater than 200. At least 10% of the chain was removed as burn-in, and runs were combined using LogCombiner v1.10.4 and downsampled to generate a final posterior distribution of 1,000 trees that was used in subsequent analyses. All parameter values have statistical uncertainty reflected in values of the 95% highest posterior density intervals. Maximum clade credibility (MCC) trees were summarized using TreeAnnotator v1.10.4 and visualized using ggtree package in R [20,21]. To identify swine transmission clusters in Italy, we used specific parameters, including a bootstrap-support threshold of 80% and a mean genetic-distance threshold of 1%, under the Cluster Picker v. 1.2.5 software [22]. Clusters were defined only if they contained at least three swine sequences, and each identified cluster was manually extended until the nearest node was supported by a bootstrap value higher than 80%. This approach ensured robust cluster identification, allowing us to effectively trace swine transmission dynamics.

### Selective pressure analysis

Non-synonymous to synonymous substitution rate (dN/dS) ratios were estimated for each segment with the single-likelihood ancestor counting (SLAC) method [23] implemented in Hyphy v.2.2.4 [24], focusing on swine samples isolated in Italy. For comparison, we also evaluated human samples isolated in the same country. The Mixed Effects Model of Evolution (MEME) method [25] in DataMonkey [26] was then used to infer the positively selected sites for each dataset (*P*<0.05).

## Results

**Fig. 1.**
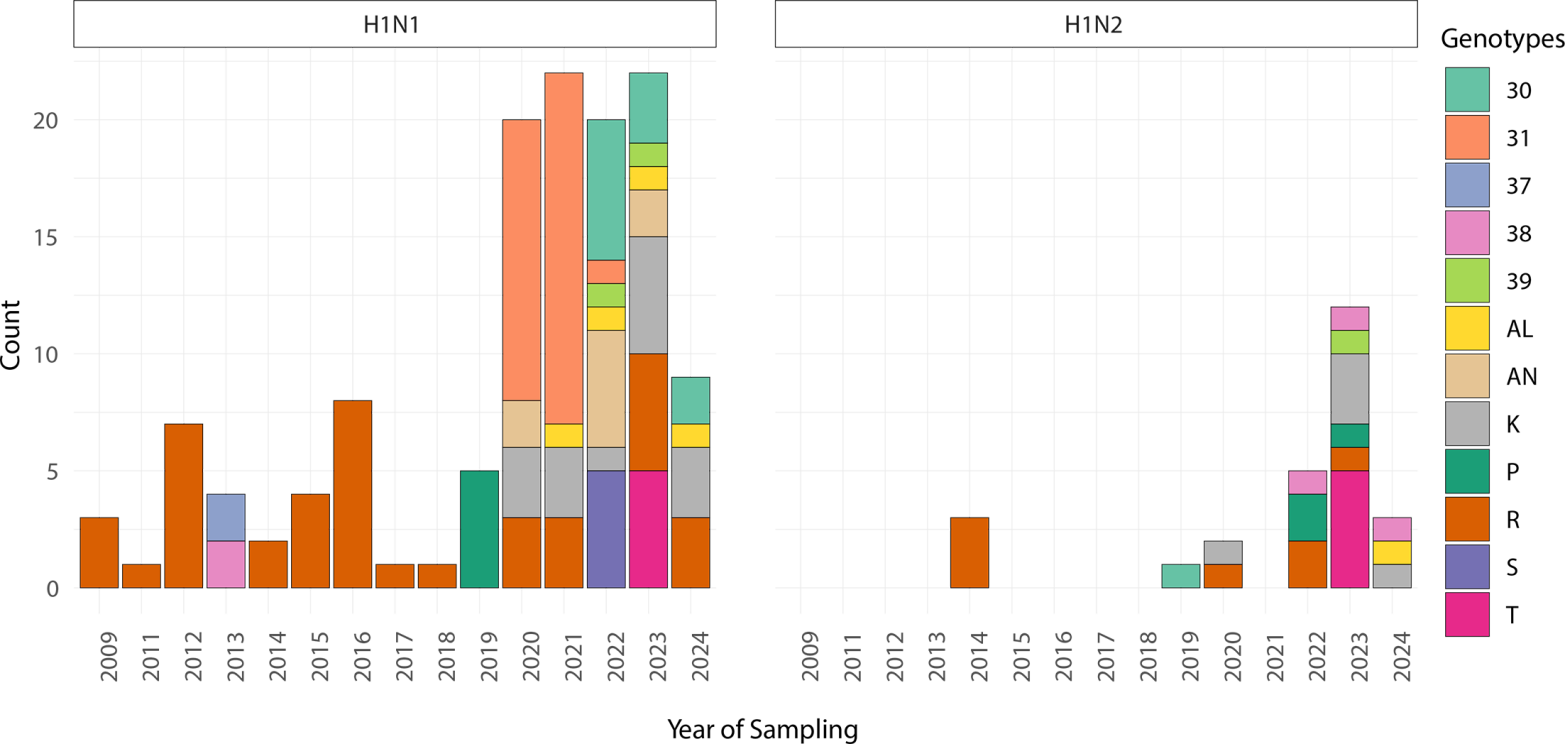
Genetic combinations detected among H1 swine influenza viruses from 2009 to 2024 in Italy. Temporal distribution of H1N1 (left) and H1N2 (right) genotypes detected over time. Each bar represents the number of cases per year, coloured by genotype (see legend on the right). Genotypes are defined as described previously (see Table 1) [10, 27].

H1N1pdm09 continues to circulate in pig herds, sustained by both repeated human-to-swine introductions and onward swine-to-swine transmission. While it has become enzootic in several regions, its evolutionary dynamics in pigs appear to differ from those of other endemic IAV-S lineages that have circulated in swine populations for decades. This may reflect differences in host adaptation, ecological fitness or immune-driven selection, although further comparative studies are needed to quantify these differences. During surveillance from 2009 to 2024, 860 different H1 strains were sequenced [10] (Fig. 1). A lower detection rate of full H1N1pdm09 strains (*n*=45) was recorded compared with IAV-S reassortant strains. Over the years, there has been an increase in reassortment events between IAV-S and H1N1pdm09 strains, in which the latter contributed all or at least one internal gene of pdm09 origin. From 2009 to 2024, we sequenced 120 IAV-S strains with H1A HA. Among them, 45 were full H1N1pdm09-derived (genotype P), while 75 were reassorting strains belonging to both H1N1 and H1N2 subtypes, each with different gene combinations [10,27]. Fig. 1 and Table 1 show the characteristics of the internal gene cassette (all genes of avian origin, all pdm origin and at least one with different origin) for each H–N combination.

**Table 1.** Total number of H1A origin strains sequenced since 2009 with the genotype assigned. Internal gene combination is shown, and nomenclature has been assigned according to previous publications [10,27]. The origin of each gene segment is classified as either pdm (derived from H1N1pdm09) or av (derived from H1N1 avian-like) for the internal protein-coding genes, while NA segments are indicated as N1av, N2g or It-N2 (Italy-specific lineage)

Subtype	Nomenclature	HA_NA_PB2_PB1_PA_NP_M_NS	Total
H1N1	31	H1pdm-1A_N1av_pdm_pdm_pdm_pdm_pdm_av	38
	37	H1pdm-1A_N1av_pdm_pdm_pdm_pdm_av_av	1
	39	H1pdm-1A_N1av_pdm_av_pdm_pdm_pdm_pdm	2
	AL	H1pdm-1A_N1pdm_pdm_pdm_pdm_pdm_pdm_av	1
	K	H1pdm-1A_N1av_av_av_av_av_av_av	3
	P	H1pdm-1A_N1pdm_pdm_pdm_pdm_pdm_pdm_pdm	45
	S	H1pdm-1A_N1av_pdm_pdm_pdm_pdm_pdm_pdm	15
H1N2	30	H1pdm-1A_It-N2_av_av_av_av_av_av	1
	38	H1pdm-1A_N2g_pdm_pdm_pdm_pdm_pdm_av	4
	AN	H1pdm-1A_N2g_av_av_av_av_av_av	1
	R	H1pdm-1A_N2g_pdm_pdm_pdm_pdm_pdm_pdm	8
	T	H1pdm-1A_N2g_pdm_pdm_pdm_pdm_pdm_pdm	1

### H1N1 swine phylodynamic reconstruction in Italy

**Fig. 2.**
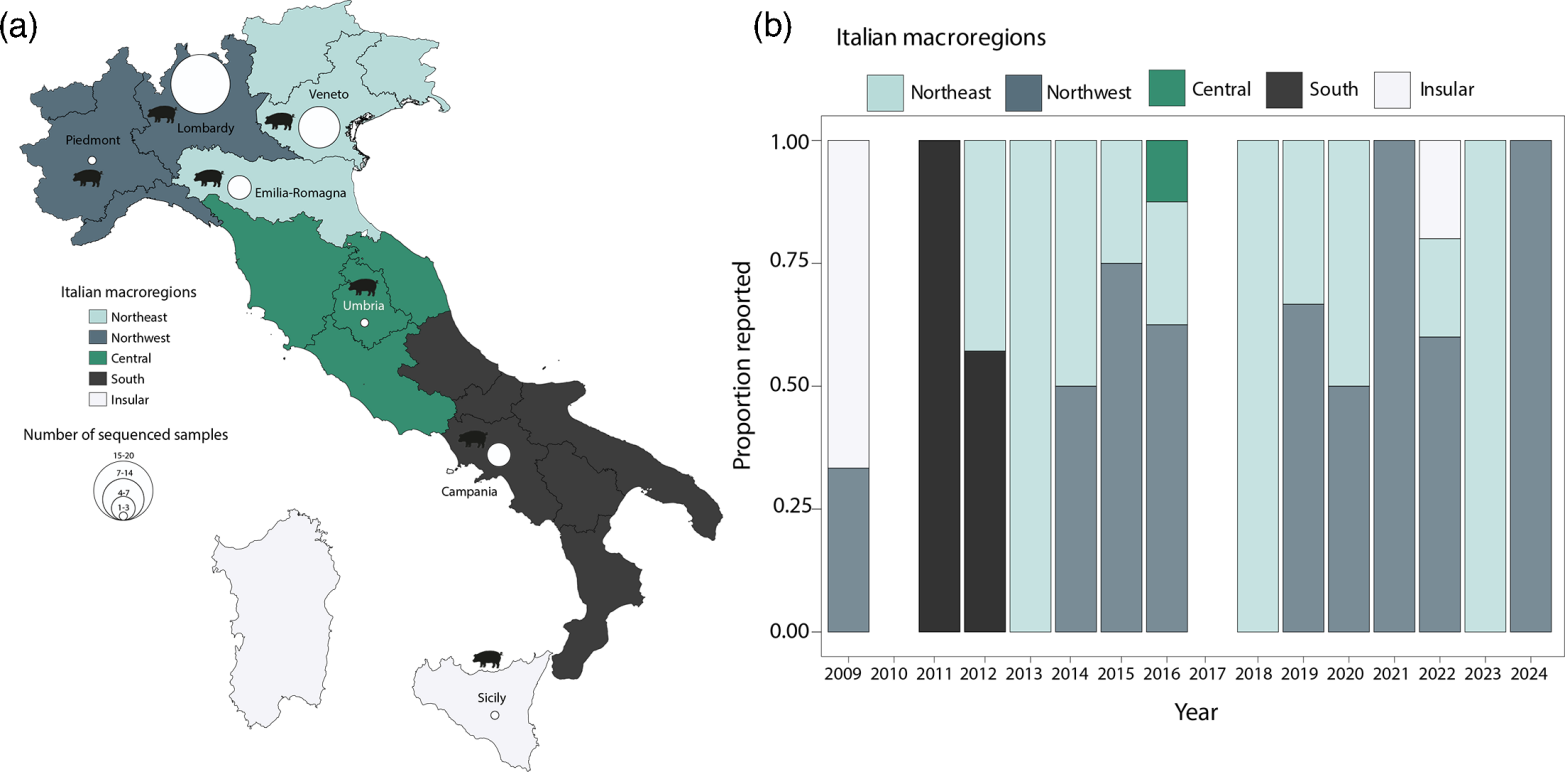
Spatial distribution and genetic diversity of H1N1pdm09 in Italian swine, 2009–2024. (a) Distribution of H1N1pdm09 swine genome sequences (*n*=45) by Italian macro-region. (**b**) Annual count of newly obtained H1N1 swine genome sequences by Italian macro-region. Colours represent different macro-regions: Northeast (light blue), Northwest (blue grey), Central (green), South (black) and Insular (white). Missing years had no genomes.

The final dataset of H1N1 swine genome sequences included samples from seven of Italy’s 20 regions. Most sequences originated from the Northwest, with Lombardy and Piedmont contributing eighteen and two sequences, respectively. In the Northeast, Veneto provided 11 sequences, and Emilia-Romagna provided five sequences. Central Italy was represented by a single sequence from Umbria. In the South, Campania contributed five sequences, while Sicily, from the Insular region, added three (Fig. 2a).

The temporal distribution of sequences (Fig. 2b) shows considerable variation in sequencing efforts over the years. These fluctuations likely reflect differences in surveillance intensity as well as the number of diagnostic-positive samples available at different times, with sequencing generally increasing during periods of higher detection. The Northeast, particularly Veneto, and the Northwest, especially Lombardy, showed increased sequencing activity after the early years of the study, possibly due to rising H1N1 case numbers or enhanced monitoring programmes. The Northwest dominated sequence collection during the middle of the study period, likely reflecting more systematic surveillance efforts. In contrast, Central Italy was underrepresented, while Southern and Insular regions contributed primarily in the earlier years. This uneven sequence distribution broadly mirrors the geographical distribution of pig density in Italy (Fig. 2a, b), which may serve as a useful proxy for surveillance expectations in areas where diagnostic data are lacking [28].

**Fig. 3.**
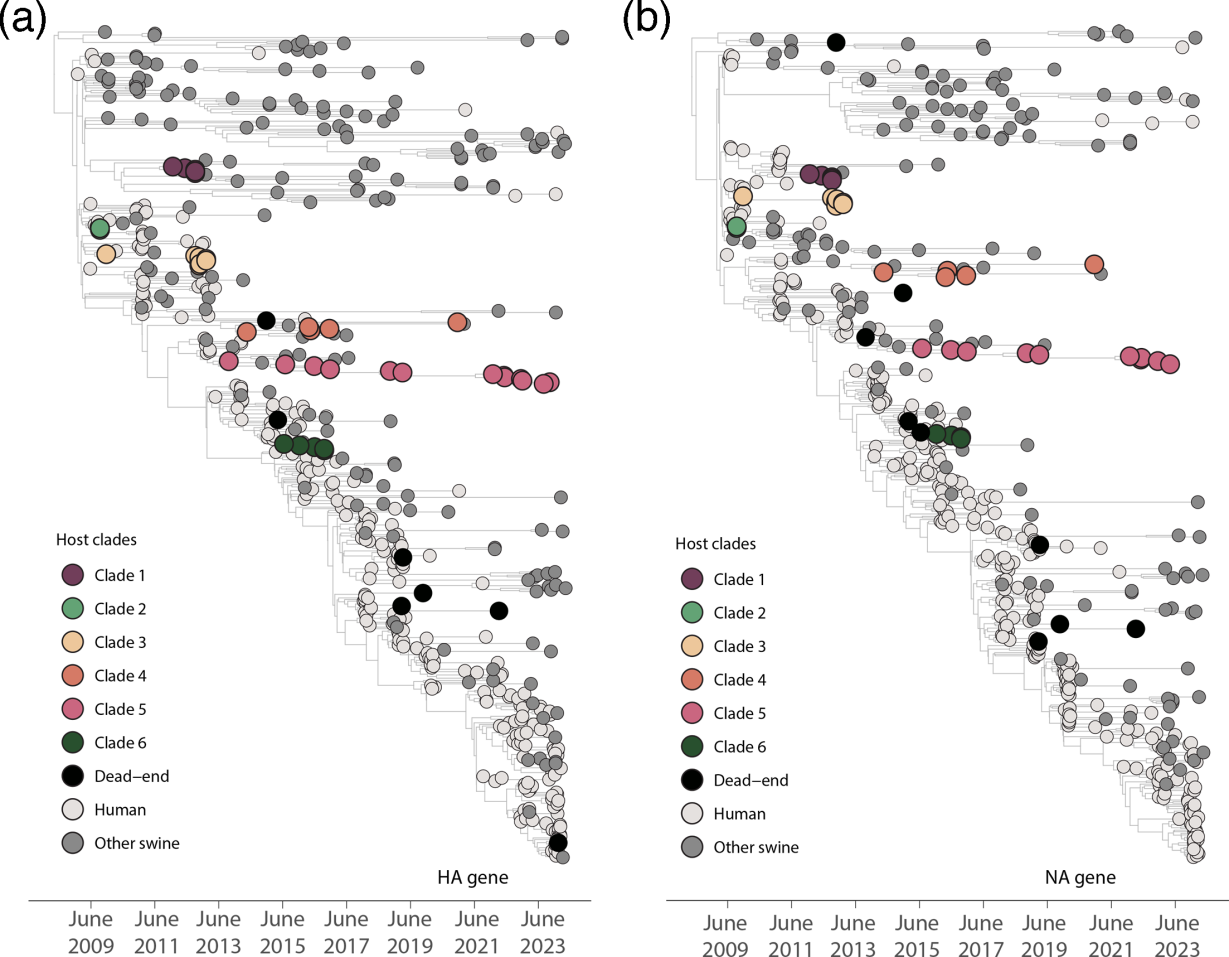
Evolutionary dynamics of Italian H1N1pdm09 HA and NA gene segments. Phylodynamic trees of the HA (**a**) and NA (**b**) genes. Tips are colour-coded according to clades: Clades 1–6 represent monophyletic groups of Italian swine isolates sampled over multiple years, suggesting sustained H1N1pdm09 transmission in Italian swine. Dead-end introductions into Italian swine, showing no evidence of onward transmission, are coloured in black. Sequences from humans and non-Italian swine populations are colour-coded with light and dark grey, respectively, as indicated in the legend. Independent introductions were inferred using 'Markov jump' counts, which measure the number of inferred transitions modelled by a continuous-time Markov chain process. These transitions occur along the branches of the phylogeny and provide a measure of gene flow.

To investigate the transmission dynamics of H1N1pdm09 swine influenza strains circulating in Italy, we conducted a comprehensive phylodynamic analysis across the eight genetic segments, as shown in Figs 3 and S1. The analysis identified six distinct clades of H1N1pdm09 circulating within Italian swine populations, marked by recurrent introductions and localized transmission patterns across all genetic segments.

Our findings revealed two primary transmission patterns: self-limited (dead-end) introductions and sustained transmission clusters. Many introductions, illustrated by singleton swine sequences or small clusters (≤2 sequences), represent isolated spillover events with limited onward spread. These are likely dead-end introductions, resulting in localized infections without broader circulation.

We estimated one introduction event from humans and at least three independent introductions from swine into Italian swine populations. These swine-derived introductions are represented by distinct monophyletic clades (Clades 1–6; Fig. 3a, b), suggesting separate transmission chains.

In contrast, several larger clades – particularly Clades 4 and 5 — showed evidence of sustained transmission over multiple years. Their monophyletic structure, composed exclusively of Italian swine sequences, suggests successful establishment and persistence of the virus within specific regions. These clusters underscore the potential for localized adaptation and long-term circulation of certain viral lineages in swine populations.

Human sequences were interspersed among swine sequences in the phylogenetic trees (Fig. 3a, b), suggesting multiple introductions of the virus from humans into swine. Directionality was formally assessed through Bayesian phylogeographic analysis using Markov jump counts, which estimated several human-to-swine transmission events. These findings were supported by high posterior probabilities (pp >0.8) in both HA and NA gene trees. Similar patterns were observed across the other gene segments (MP, PA, PB2, PB1, NP and NS), where distinct swine-derived monophyletic clades indicated onward transmission following initial introductions.

Beyond the evidence of interspecies transmission, the phylodynamic analysis revealed important insights into the spatial and genomic diversification of these swine-adapted clades. Distinct regional distributions of viral clades were observed across different gene segments (Fig. S2). Clade 1 was predominantly detected in the South (black bars) and was present in nearly all gene segments, indicating a geographically restricted yet persistent circulation in this region. Clade 2 was mostly found in the Insular region (light grey bars), with limited detection across HA and NP, suggesting a localized and possibly short-lived introduction. Clade 3 showed modest representation, mainly in the Central (green) and Northwest (dark blue) regions, particularly in NP, NS and PB1 segments, suggesting limited spread. Clade 4 displayed a broader regional distribution, with sequences identified in the Northeast (light blue), Northwest and Central regions across several gene segments – especially PB1, PB2 and MP – indicating ongoing circulation across northern and central Italy. Clade 5 was among the most widely distributed and genetically represented, appearing across all regions and segments, with particularly high counts in the Northeast and Northwest. This suggests both extensive spatial spread and strong local persistence. Finally, Clade 6, though less frequent, was found mainly in the Northwest and South, pointing to more recent or localized introductions with some regional overlap.

**Fig. 4.**
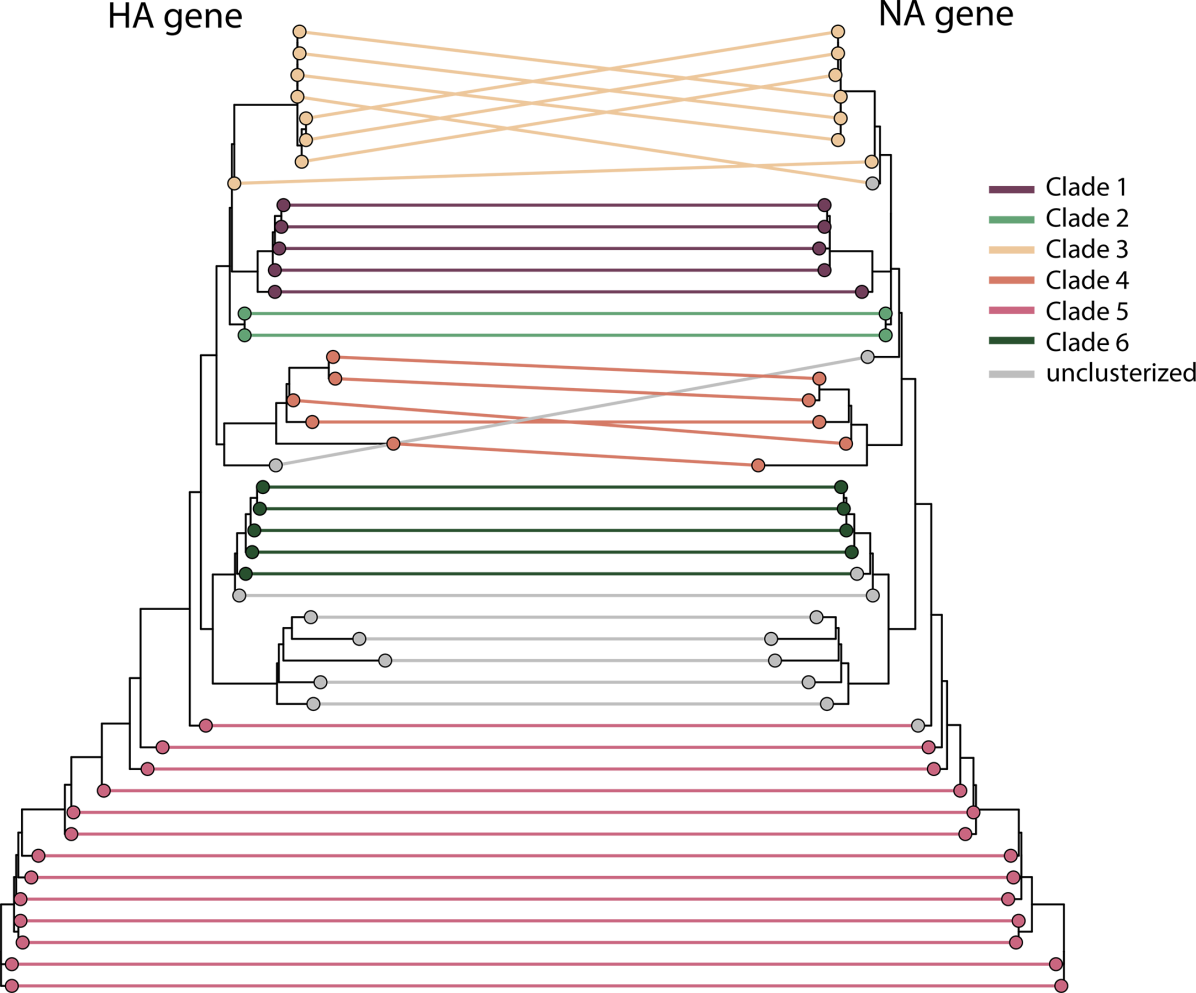
Tanglegram of swine Italian H1N1pdm09 HA and NA strains. Corresponding taxa in the two trees are connected by lines. The tips are coloured according to clade membership. The connecting lines are coloured according to the corresponding HA gene clade.

Additionally, reassortment events between the HA, NA and other genome segments of strains circulating in Italian swine were evaluated through phylogenetic inference (Figs 4, S3 and S4, available in the online Supplementary Material).

Our analysis revealed consistent topologies for HA and NA in Clades 1, 2, 5 and 6. However, we identified two incongruences: one sequence from outside NA Clade 3 grouped within HA Clade 3, and another sequence from outside NA Clades 1, 2, 3, 4 and 6 was found within HA Clade 5. When comparing HA and NA with the other six gene segments, consistent topology and clade assignment were observed only for Clade 2 (Fig. S3). The greatest number of incongruences was seen in the MP gene comparison, where all clades except Clade 2 showed inconsistencies (Fig. S3c, i). Most discordances occurred in sequences assigned to a clade in one gene but were unclassified in another, but with a similar topology, particularly involving Clades 4, 5 and 6 (Fig. S3). Mismatched pairings were observed between sequences classified as Clade 5 in one gene and those from Clade 4 in another. This was most notable in comparisons between HA and NP, PB1 and PA, as well as NA and NP, MP and PA (Fig. S3a, d, f, g, i, l). Comparisons of HA and NA with the other six gene segments revealed a greater extent of reassortment, while comparing the six segments among themselves showed fewer differences. Notably, significant incongruences were found between MP clades and other gene clades (mostly Clades 1 and 4), as well as between PB2 clade 4 and the remaining gene Clade 4 with a discordant topology (Fig. S4b–d, h, i, l, m, n). These findings confirm that reassortment is taking place among H1N1pdm09 viruses circulating in Italian swine, indicating ongoing genetic exchange between different viral strains.

### Evolutionary dynamics of H1N1pdm09 in swine populations across Italy

**Fig. 5.**
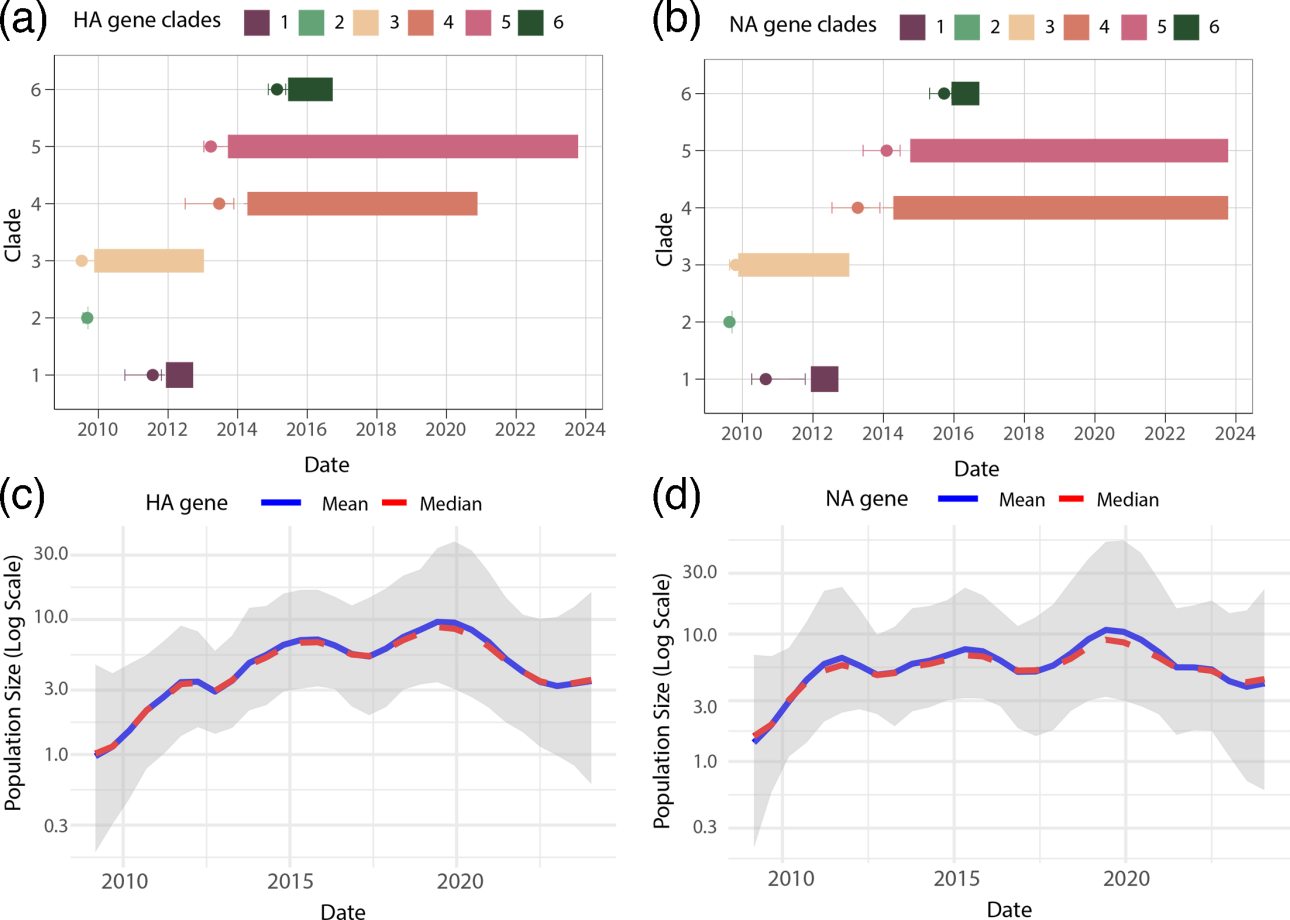
Temporal evolution and population dynamics of H1N1pdm09 HA and NA gene clades in swine populations. (**a**) Temporal distribution of HA gene clades (Clades 1–6) in H1N1pdm09 swine influenza virus populations in Italy. The *x*-axis represents the time period from 2010 to 2024, while the *y*-axis shows the identified clades. Each clade is depicted with coloured bars indicating the span of its detection, with markers denoting the median time to the MRCA; (**b**) Temporal distribution of NA gene clades (Clades 1–6), following the same structure as panel (**a**). (c) Bayesian Skyline plot showing the population size dynamics for the HA gene over time, with effective population size (log scale) on the *y*-axis and time on the *x*-axis. The solid blue line represents the mean estimate, while the red line denotes the median estimate. The shaded area reflects the 95% HPD interval. (**d**) Population size dynamics for the NA gene, following the same structure as panel (**c**). MRCA, most recent common ancestor.

To better understand the evolutionary dynamics of H1N1pdm09 in Italian swine populations, we performed a comprehensive phylodynamic analysis (Figs 5, S5 and S6). MCC trees were inferred for all HA (*n*=6) and NA (*n*=6) clades identified in the ML phylogenies (Fig. 5a, b). The resulting topologies were consistent with those obtained by ML inference and allowed us to estimate the timing of viral introductions into swine. Specifically, three of the six HA and NA clades (Clades 1–3) showed ancestral nodes dating back to 2009–2011, suggesting that multiple persistent lineages likely originated from early human-to-swine transmission events during the initial years of the H1N1pdm09 pandemic. The remaining clades (Clades 4–6) emerged later, possibly resulting from subsequent introductions or diversification within swine. These patterns indicate a combination of early establishment and ongoing introduction events, shaping the viral population structure in swine over time.

Bayesian reconstructions indicate that HA Clades 1, 3, 4 and 5 likely originated before 2014, with detection periods ending in 2013, 2013, 2021 and 2023, respectively (Fig. 5a). A similar trend was observed in the NA gene for Clades 1, 3, 4 and 5 (Fig. 5b), with Clades 4 and 5 persisting the longest – up to 2023. Notably, Clade 5 circulated in swine populations for ~9 years, underscoring the ability of certain viral lineages to persist over extended periods. To further explore transmission dynamics, we estimated the effective population size (Ne) using the GMRF Skyride coalescent model (Fig. 5c, d). In this context, Ne reflects relative genetic diversity over time, which is influenced by transmission intensity and population structure, rather than the actual number of animals in the swine population. Therefore, while Ne is not a direct proxy for census population size, it serves as an informative indicator of viral spread and persistence. Our results reveal an initial exponential increase in Ne for both HA and NA genes until around 2012, coinciding with the early phase of human-to-swine transmission and viral establishment in swine populations. This was followed by a stabilization phase, suggesting a dynamic equilibrium between ongoing swine-to-swine transmission and lineage extinction. From 2020 onward, a slight decline in Ne was observed, which may reflect reduced transmission, effective control measures or decreased sampling – especially during the Coronavirus Disease 2019 (COVID-19) pandemic. Additionally, our analysis suggests possible cryptic transmission in some clades, where sequences appear to have circulated for a period prior to broader detection. While Clade 5 shows sustained circulation over ~9 years, its detection closely follows its estimated most recent common ancestor (TMRCA) (Fig. 5a, b), indicating early capture by surveillance. In contrast, for certain other clades (e.g. Clade 6), the first detection appears to occur well after the inferred time of introduction, suggesting delays in detection and underscoring the need to strengthen early surveillance efforts to reduce undetected viral spread.

### Evolutionary pressures shaping H1N1pdm09 within the Italian swine populations

**Fig. 6.**
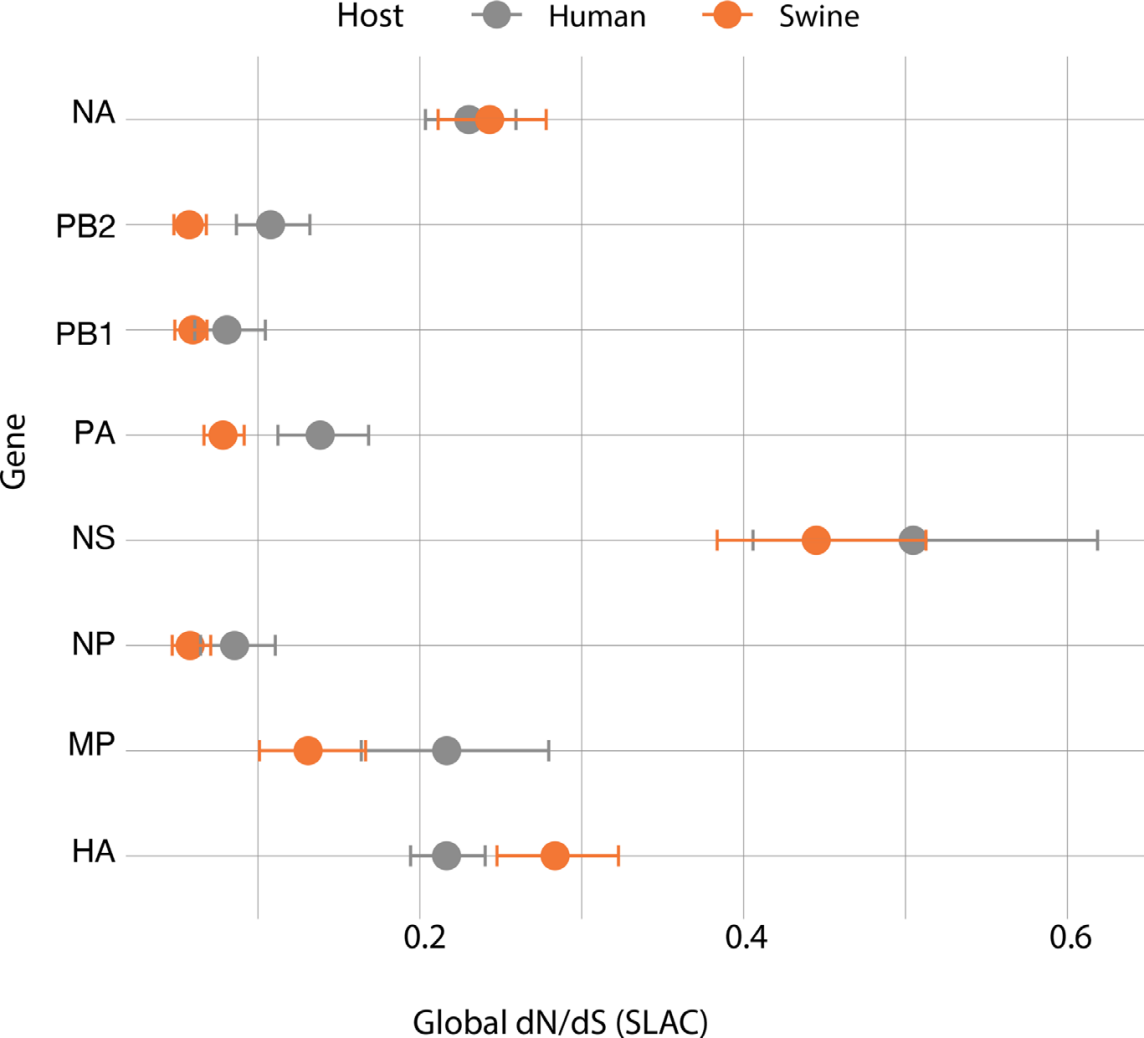
Selective pressure on H1N1pdm09 gene segments in Italian swine and human populations. Global dN/dS ratios are shown for eight gene segments (NA, PB2, PB1, PA, NS, NP, MP and HA) of H1N1pdm09 circulating in Italian swine (orange) and human (grey) populations, estimated using the SLAC method. The *x*-axis represents the global dN/dS ratio, and the *y*-axis lists the gene segments. Circles represent the dN/dS estimates, with horizontal bars indicating the confidence intervals. Ratios below 1 suggest purifying selection.

We also investigated the selective pressures acting on the H1N1pdm09 virus by comparing dN/dS ratios across different gene segments in both swine and human hosts (Fig. 6).

The dN/dS ratio is an important measure of evolutionary pressure: values below 1 indicate purifying selection (selection against non-synonymous mutations), values around 1 suggest neutral evolution, and values above 1 indicate positive selection (favouring non-synonymous mutations). The analysis reveals that all H1N1pdm09 genes are subject to purifying selection in both swine and human hosts, with dN/dS ratios well below 1. These results suggest that these genes are mostly evolving via genetic drift and that non-synonymous mutations are largely deleterious, being selected against in both species. However, some differences between swine and human hosts were observed. The HA gene is the only one exhibiting a higher dN/dS ratio in swine (~0.28) compared with humans (~0.22), suggesting relaxed selective pressure or ongoing adaptation in swine relative to the stronger purifying selection observed in humans, based on analysis of 185 Italian human H1N1pdm09 strains collected between 2009 and 2024 from GISAID. The overall trend in the dN/dS ratio was similar in both species across all genes, likely due to multiple introductions from humans to swine. These findings highlight that, while purifying selection dominates the evolutionary dynamics of H1N1pdm09 virus in both hosts, the viral attachment protein HA, which is the primary target of the humoral immune response, may experience weaker selection constraints, particularly in swine. This shows that swine populations may act as reservoirs occasionally for viral evolution, with the potential for genetic changes that may influence transmission and pathogenicity [29].

## Discussion

The dN/dS ratio is an important measure of evolutionary pressure: values below 1 indicate purifying selection (selection against non-synonymous mutations), values around 1 suggest neutral evolution, and values above 1 indicate positive selection (favouring non-synonymous mutations). Through the analysis of 45 genome sequences from different Italian regions, we gained a deeper understanding of the virus’s evolutionary patterns and its geographical spread within the country. The regional distribution of sequences highlights the uneven nature of surveillance efforts across Italy. Seventy per cent of the pig population is concentrated in the northern regions of Lombardy, Veneto, Piedmont and Emilia-Romagna, where industrial-scale pig farming is predominant [30]. This explains the greater number of sequences obtained from these areas, particularly Lombardy, which contributes significantly to national pig production and accounts for a substantial proportion of sequencing efforts. Conversely, regions with smaller pig populations are naturally expected to yield fewer viral sequences, which may more accurately reflect the real prevalence of circulating strains in these areas.

Addressing this balance in surveillance is key for ensuring a comprehensive understanding of the epidemiology of H1N1pdm09 across the country, as the smaller number of sequences from certain regions may still provide valuable insights into local viral circulation patterns. Strengthening surveillance across all regions, including areas with fewer pigs, could enhance early detection of emerging strains and support a more proactive approach to influenza monitoring in Italy.

Our phylodynamic analysis revealed a complex pattern of multiple independent introductions of H1N1pdm09 into Italy, resulting in both short-lived (dead-end) transmission events and sustained swine-specific transmission clusters. The identification of six distinct clades, with long-term circulation observed in Clades 1, 3, 4 and 5, suggests that some lineages became established in swine populations, while others likely reflect periodic reintroductions from external sources. We distinguish reintroduction from sustained transmission based on tree topology and temporal structure: clades with short branch lengths, limited geographic spread and no onward transmission are consistent with reintroduction events followed by local extinction (e.g. Clades 2 and 6). In contrast, clades showing monophyletic structure, continuity across multiple years and regional clustering, such as Clades 3 and 5, indicate sustained circulation within swine (Fig. 5a, b). Some of these reintroductions may have originated from human populations, while others could represent cross-border incursions or introductions from unsampled swine reservoirs. These patterns emphasize the importance of continuous genomic surveillance to detect both reintroduction events and locally evolving lineages [31,32]. Moreover, our findings reveal important evolutionary dynamics, including reassortment and genetic diversification, shaping the landscape of H1N1pdm09 in Italian swine. Consistent phylogenetic topologies for the HA and NA genes in Clades 1, 2, 5 and 6 suggest relatively stable evolutionary histories for these surface proteins. However, marked incongruences in other segments – particularly outside established NA clades – point to frequent reassortment events. Notably, discrepancies between HA and NA trees in Clades 3 and 5 indicate genetic exchanges, likely giving rise to novel viral genotypes with potentially altered phenotypes. Additional discordances between internal gene segments (e.g. PA versus MP; PB2 versus MP and NS) further highlight the modular nature of influenza virus evolution. These reassortments may impact viral fitness, transmissibility or antigenicity, posing ongoing risks to swine health and, potentially, to human populations.

The selective pressure analysis underscores the role of host-specific adaptation in shaping the evolutionary trajectories of IAVs. As commonly observed in influenza viruses, all gene segments are predominantly under strong purifying (negative) selection in both swine and human hosts, consistent with the need to maintain essential viral functions. This pattern aligns with previous studies and reflects the relatively conserved nature of internal genes and structural constraints. Most segments – including NA, PB2, PB1 and PA – exhibited similarly low dN/dS ratios in both hosts. However, a modest difference was observed in the HA gene, where swine viruses showed a slightly higher dN/dS ratio (~0.28) compared with human viruses (~0.22). While both values remain well below 1, indicating purifying selection, the relatively weaker selective pressure in swine may suggest greater tolerance for amino acid substitutions in HA gene, potentially due to less immune pressure or differences in vaccine coverage.

This observation could reflect the immunological context of swine populations, where vaccination strategies are not systematically applied and herd immunity is often heterogeneous. Such conditions may favour transient diversification or relaxed purifying selection at antigenic sites, as the virus circulates in partially immune herds. Conversely, the higher and more uniform immune pressure in humans likely constrains HA evolution more strongly. Although our dataset did not reveal clear evidence of positive selection, this pattern may still reflect antigenic drift occurring under suboptimal immune control. Future studies incorporating antigenic site mapping and broader surveillance could help determine whether specific substitutions in swine HA correspond to known antigenic epitopes [33].

The use of a vaccine containing H1N1pdm2009 strains in pig populations may also have contributed to this trend [33]. It is important to note that, while purifying selection dominates, episodic or site-specific positive selection – particularly at antigenic sites of the HA or NA proteins – can occur during host adaptation or in response to immune pressure. In this study, however, we did not detect clear signals of positive selection, which may reflect limited immune-driven evolution in the swine population during the sampling period. Nonetheless, the observed patterns highlight the evolutionary flexibility of HA in swine and the need for ongoing surveillance.

Our findings also reinforce the importance of a One Health approach [34], integrating human, animal and environmental health sectors to tackle the multifaceted nature of viral transmission. The evidence of rare human-to-swine spillovers, coupled with the persistence of swine-adapted lineages, underscores the need for coordinated monitoring of both human and swine populations to fully capture the interspecies dynamics of the virus and to mitigate future zoonotic spillovers. Italy’s significant role in global influenza monitoring is evident, given its large swine population and the ongoing risk of viral reassortment with human or avian influenza strains. By linking veterinary surveillance with public health initiatives, we can better anticipate and respond to viral transmission events that may have pandemic potential. The detection of multiple introductions and sustained viral transmission in Italy’s swine populations further underscores the need for ongoing, widespread surveillance efforts. Increased surveillance is crucial for ensuring a more complete epidemiological understanding. Furthermore, international collaboration is essential to track the global movement of influenza strains and implement coordinated strategies to manage the spread of zoonotic diseases.

In conclusion, this study provides a detailed characterization of the genetic diversity, reassortment patterns and selective pressures shaping the evolution of H1N1pdm09 in Italian swine populations. While not quantifying transmission routes directly, our findings offer important insights into the interplay between human and swine hosts and reveal evidence of both repeated introductions and sustained circulation of specific viral clades. The results underscore the value of continuous genomic surveillance – especially in under-sampled regions – to detect emerging lineages and assess associated risks. Through collaborative efforts and phylodynamic inference, we can enhance our understanding of the evolutionary dynamics underlying swine influenza and improve preparedness for future zoonotic threats. Adopting a One Health approach remains essential to developing integrated and effective strategies to monitor and mitigate influenza virus transmission in animal populations.

## Limitation

While our study provides important insights into the transmission dynamics and genetic diversity of H1N1pdm09 in Italian swine populations, several limitations should be noted. First, our sample size, though representative, may not capture the full spectrum of genetic diversity across all Italian swine farms, potentially limiting the generalizability of our findings. Additionally, our analysis is constrained by the inherent limitations of dynamic modelling, which relies on assumptions that may not account for all ecological and epidemiological complexities influencing virus transmission. Finally, while selective pressure analysis offers valuable perspectives on evolutionary dynamics, interpretation should be approached with caution, as certain selective signals might be influenced by sampling biases or localized viral adaptations. Addressing these limitations in future studies could help refine our understanding of H1N1pdm09 spread and persistence in swine populations, further enhancing our preparedness against zoonotic influenza risks.

## Supplementary material

10.1099/jgv.0.002174 Uncited Supplementary Material 1.

10.1099/jgv.0.002174 Uncited Table S1.

## Abbreviations

COVID-19: Coronavirus Disease 2019
dN/dS: non-synonymous to synonymous substitution rate
FAO: Food and Agriculture Organization
GISAID: Global Initiative on Sharing All Influenza Data
HA: haemagglutinin
HPD: highest posterior density
IAV: influenza A virus
IAV-S: influenza A viruses in swine
MCC: maximum clade credibility
MCMC: Markov Chain Monte Carlo
MEME: Mixed Effects Model of Evolution
ML: maximum-likelihood
NA: neuraminidase
NS: non-structural
RT-PCR: real-time PCR
SLAC: single-likelihood ancestor counting
WOAH: World Organisation for Animal Health
