# Supplementary material for: From North to South: transmission dynamics of H1N1pdm09 swine influenza A viruses in Italy
Source: J Gen Virol. 2025 Nov 13;106(11):002174. doi: 10.1099/jgv.0.002174 (PMC12614361; doi:10.1099/jgv.0.002174)
Supplement: Uncited Supplementary Material 1. [file jgv-106-02174-s001.pdf]

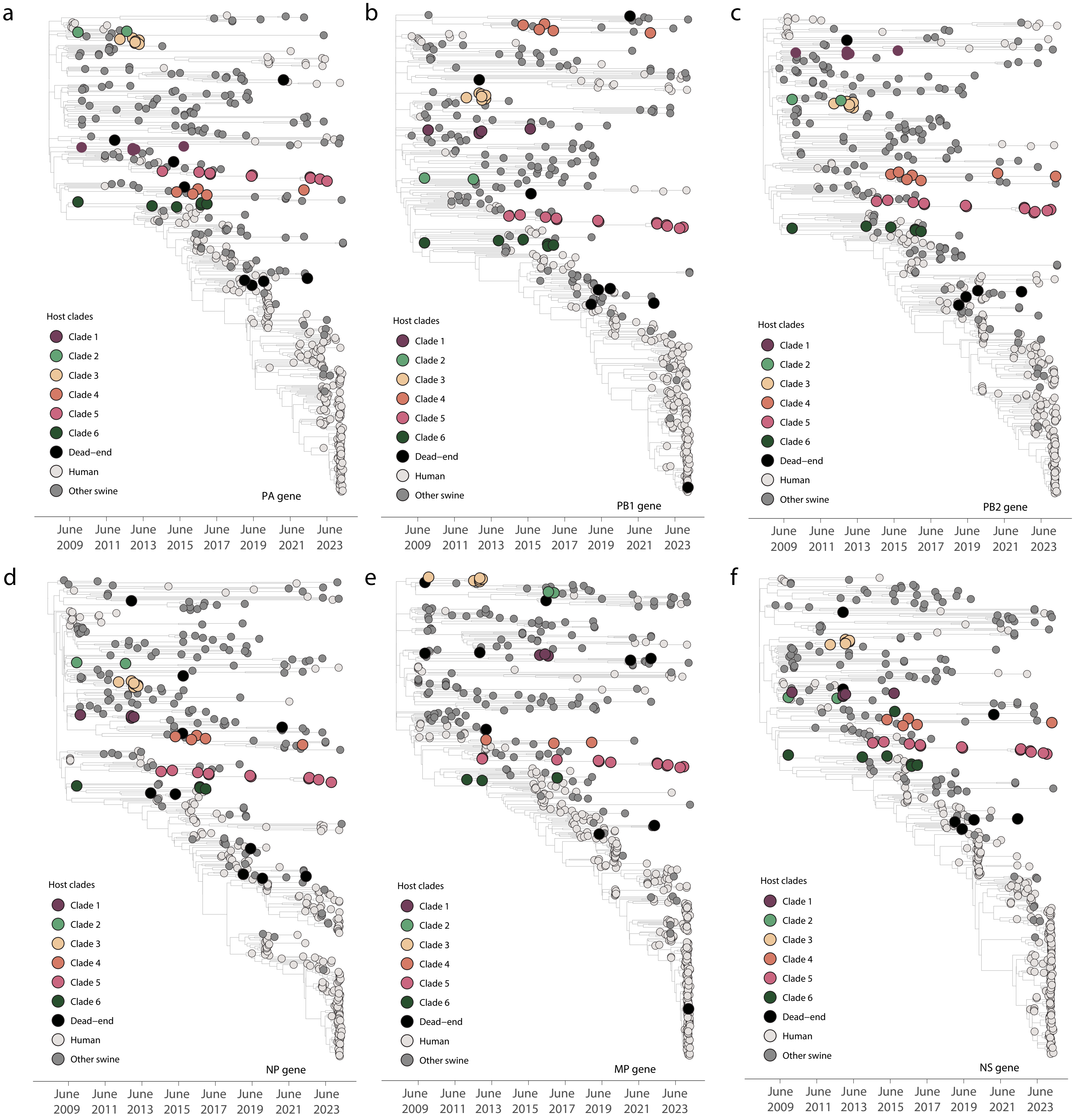

Figure S1. Evolutionary dynamics of Italian H1N1pdm09 PA, PB1, PB2, NP, MP and NS strains. Phylodynamic maximum clade credibility trees of the PA (a), PB1 (b), PB2 (c), NP (d), MP (e), and NS (f) genes. Tips are color-coded according to clades: Clades 1 to 6 represent monophyletic groups of Italian swine isolates sampled over multiple years, indicating introduction and sustained H1N1pdm09 transmission in Italian swine. Dead-end introductions, which show no evidence of onward transmission, are colored in black. Sequences from humans are color-coded in light gray, and sequences from other swine populations are shown in dark gray, as indicated in the legend. Independent introductions were inferred using 'Markov jump' counts, which measure the number of inferred transitions modeled by a continuous-time Markov chain process. These transitions occur along the branches of the phylogeny and provide a measure of gene flow.

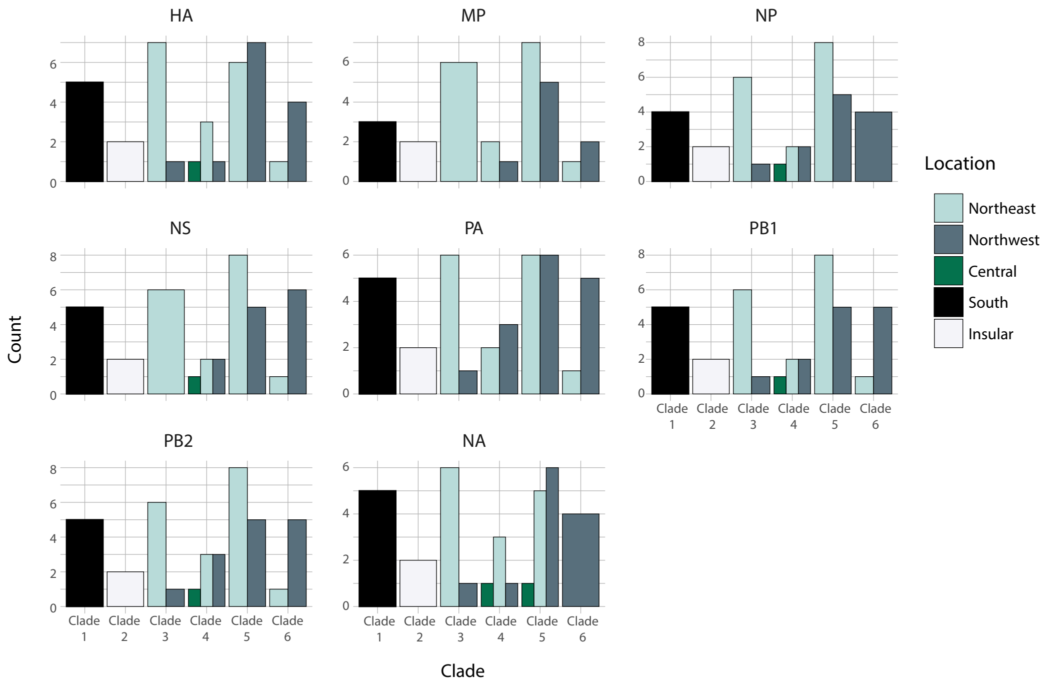

Figure S2. Regional composition of the six viral clades across various genetic segments. The Italian regions are grouped by macroregion and each bar is colored according to the legend on the right.

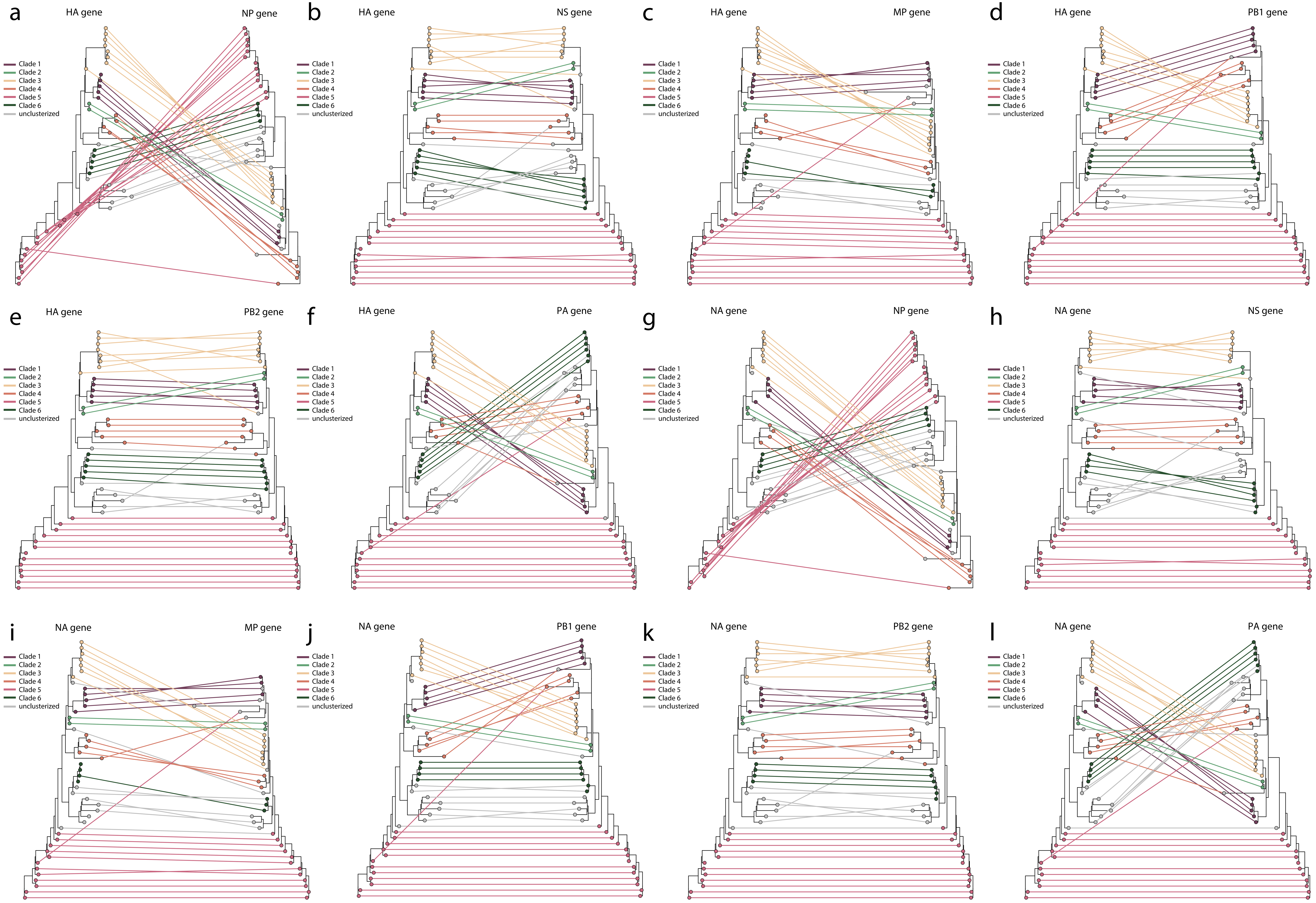

Figure S3. Tanglegrams of swine Italian H1N1pdm09 HA and NA strains with the six internal genes (NS, NP, MP, PA, PB1 and PB2). Corresponding taxa in the two trees are connected by a line. The tips are colored according to the clade membership. The connecting lines are colored by the left-side gene corresponding clade. The legend is on the left-side of the each tanglegram.

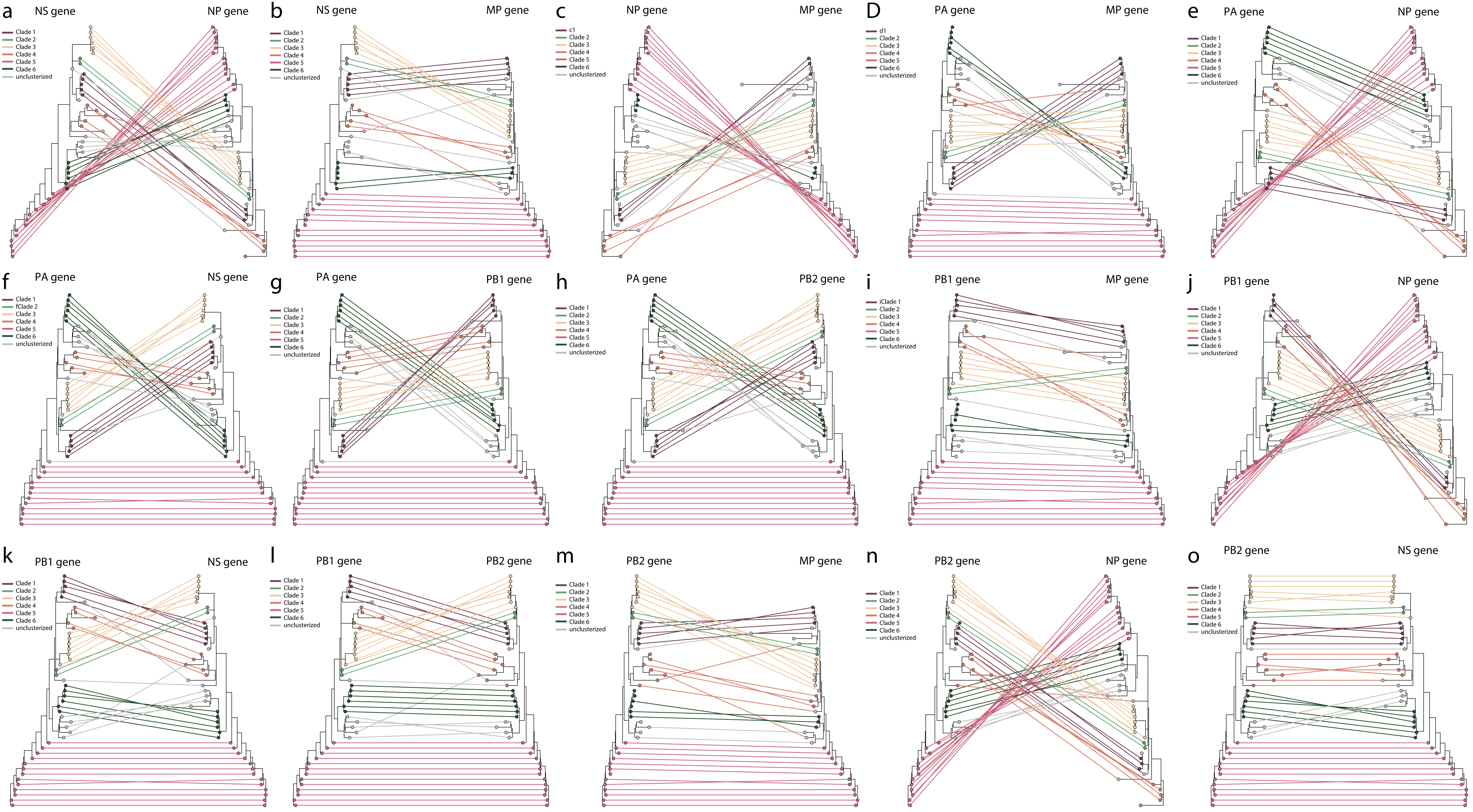

Figure S4. Tanglegrams of swine Italian H1N1pdm09 internal genes (NS, NP, MP, PA, PB1 and PB2). Corresponding taxa in the two trees are connected by a line. The tips are colored according to the clade membership. The connecting lines are colored by the left-side gene corresponding clade. The legend is on the left-side of the each tanglegram.

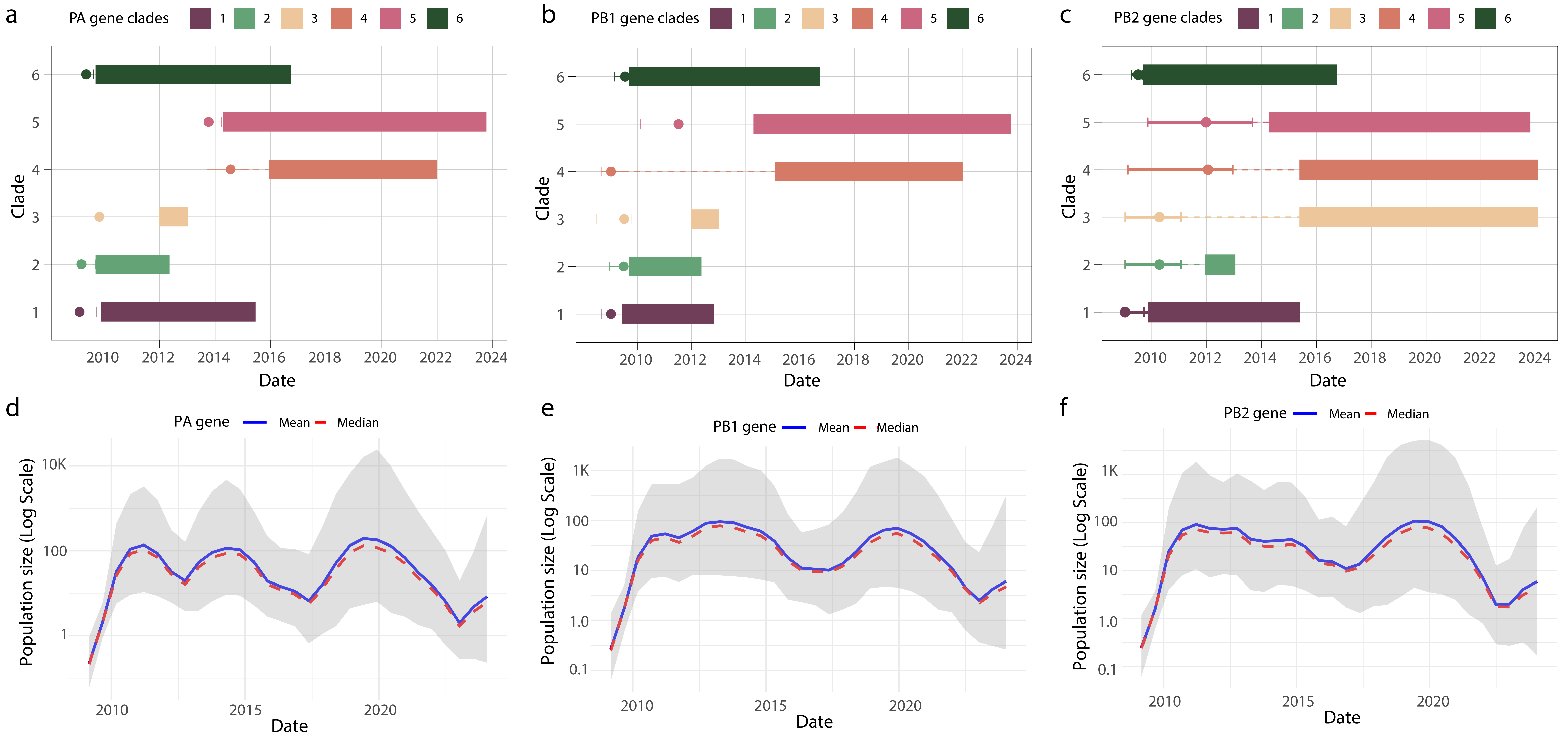

Figure S5. Temporal Evolution and Population Dynamics of H1N1pdm09 PA, PB1 and PB2 Gene Clades in Swine Populations. a) Temporal distribution of HA gene clades (Clades 1–6) in H1N1pdm09 swine influenza virus populations in Italy. The x-axis represents the time period from 2010 to 2024, while the y-axis shows the identified clades. Each clade is depicted with colored bars indicating the span of its detection, with markers denoting the median time to the most recent common ancestor (MRCA); b) Temporal distribution of NA gene clades (Clades 1–6), following the same structure as panel (a), showing the persistence and detection of clades from 2010 to 2024; c) Bayesian Skyline plot showing the population size dynamics for the HA gene over time, with the effective population size (log scale) on the y-axis and the time on the x-axis. The solid blue line represents the mean estimate, while the red line denotes the median estimate. The shaded area reflects the 95% highest posterior density (HPD) interval; d) Population size dynamics for the NA gene, following the same structure as panel (c), showing changes in effective population size (Ne) from 2010 to 2024, with the shaded area representing the 95% HPD interval.

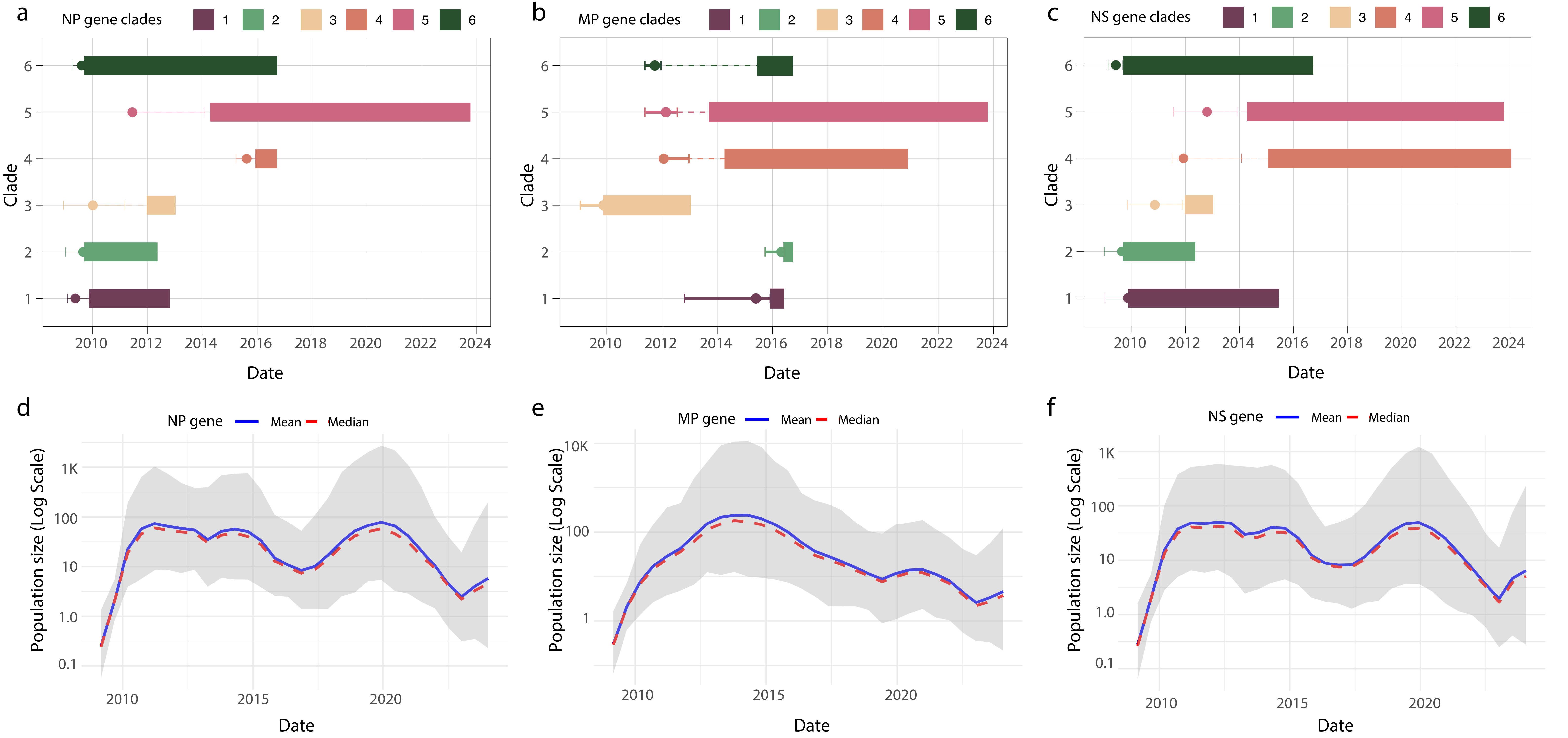

Figure S6. Temporal Evolution and Population Dynamics of H1N1pdm09 NP, MP and NS Gene Clades in Swine Populations. a) Temporal distribution of HA gene clades (Clades 1–6) in H1N1pdm09 swine influenza virus populations in Italy. The x-axis represents the time period from 2010 to 2024, while the y-axis shows the identified clades. Each clade is depicted with colored bars indicating the span of its detection, with markers denoting the median time to the most recent common ancestor (MRCA); b) Temporal distribution of NA gene clades (Clades 1–6), following the same structure as panel (a), showing the persistence and detection of clades from 2010 to 2024; c) Bayesian Skyline plot showing the population size dynamics for the HA gene over time, with the effective population size (log scale) on the y-axis and the time on the x-axis. The solid blue line represents the mean estimate, while the red line denotes the median estimate. The shaded area reflects the 95% highest posterior density (HPD) interval; d) Population size dynamics for the NA gene, following the same structure as panel (c), showing changes in effective population size (Ne) from 2010 to 2024, with the shaded area representing the 95% HPD interval.
